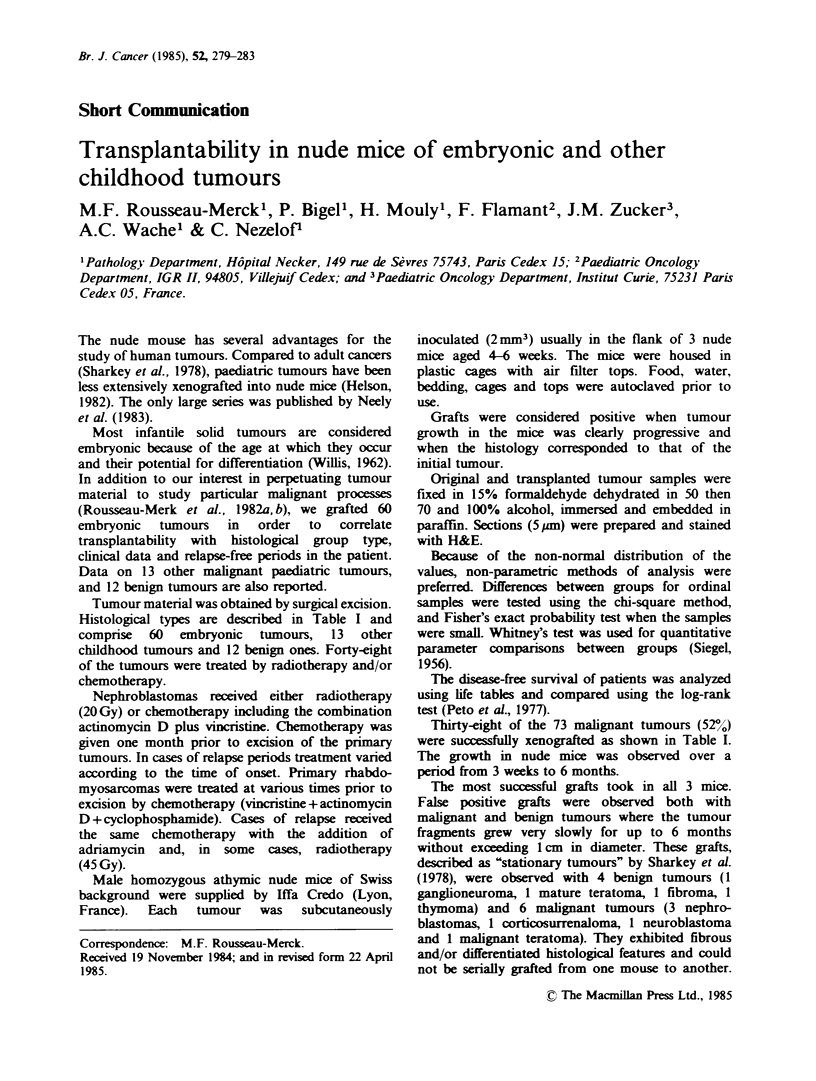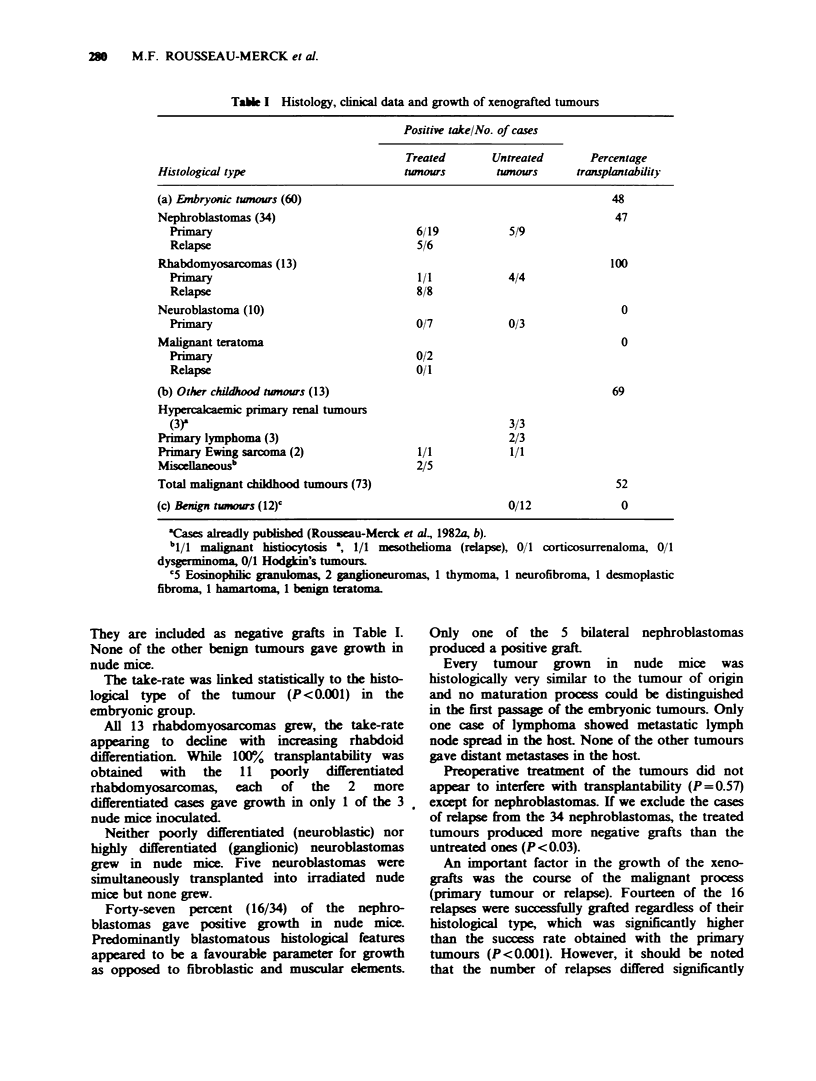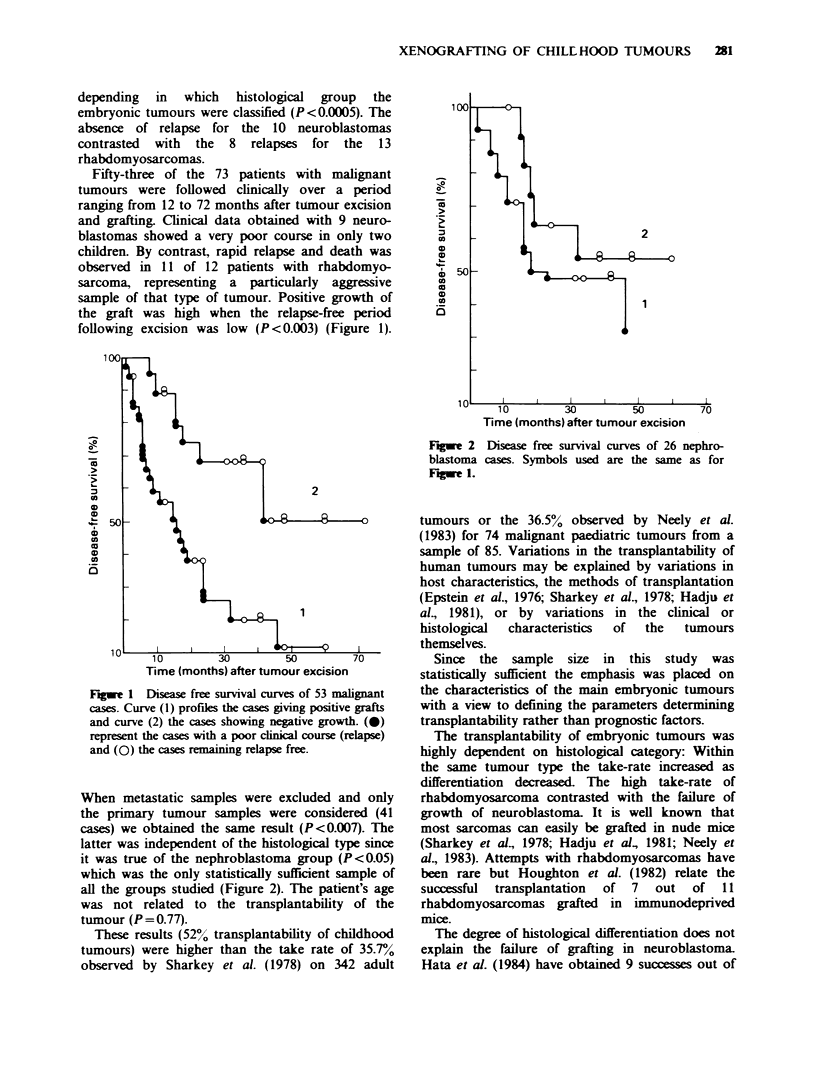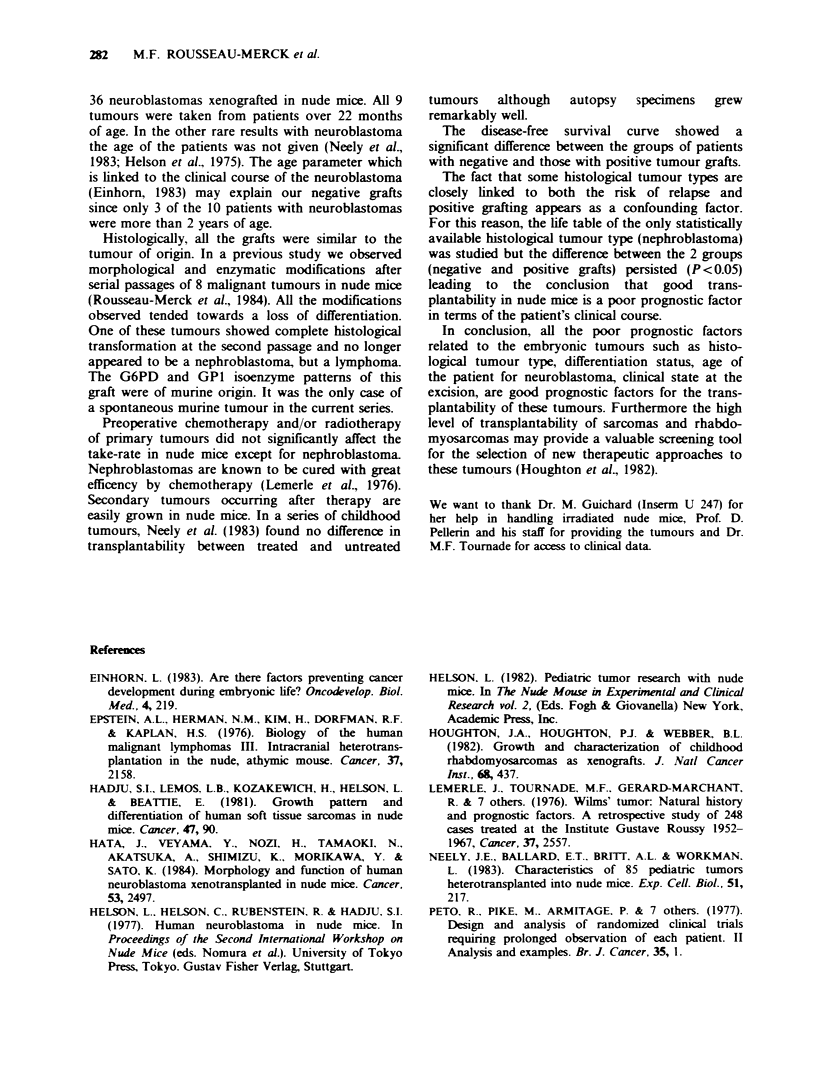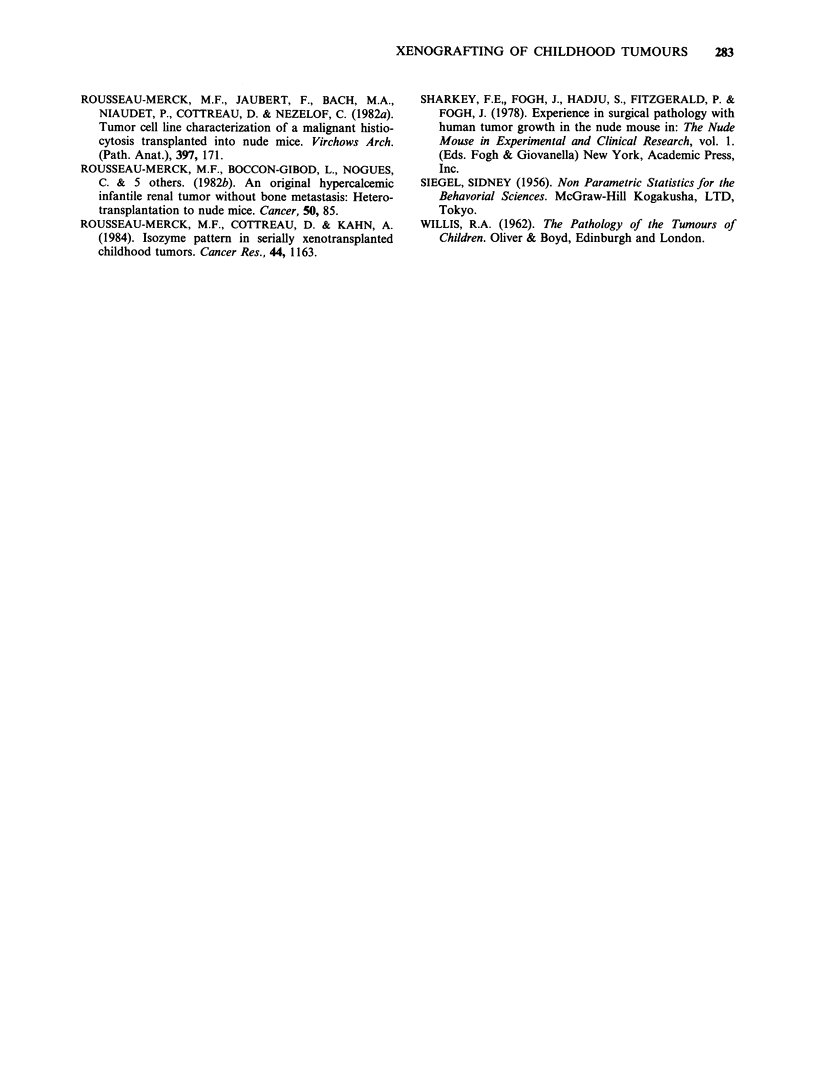# Transplantability in nude mice of embryonic and other childhood tumours.

**DOI:** 10.1038/bjc.1985.189

**Published:** 1985-08

**Authors:** M. F. Rousseau-Merck, P. Bigel, H. Mouly, F. Flamant, J. M. Zucker, A. C. Wache, C. Nezelof


Br. J. Cancer (1985), 52, 279–283

**Short Communication**

### Transplantability in nude mice of embryonic and other childhood tumours

M.F. Rousseau-Merck[1], P. Bigel[1], H. Mouly[1], F. Flamant[2], J.M. Zucker[3], A.C. Wache[1] & C. Nezelof[1]

[1]*Pathology Department, Hôpital Necker, 149 rue de Sèvres 75743, Paris Cedex 15;* [2]*Paediatric Oncology Department, IGR II, 94805, Villejuif Cedex; and* [3]*Paediatric Oncology Department, Institut Curie, 75231 Paris Cedex 05, France.*

The nude mouse has several advantages for the study of human tumours. Compared to adult cancers (Sharkey *et al.*, 1978), paediatric tumours have been less extensively xenografted into nude mice (Helson, 1982). The only large series was published by Neely *et al.* (1983).

Most infantile solid tumours are considered embryonic because of the age at which they occur and their potential for differentiation (Willis, 1962). In addition to our interest in perpetuating tumour material to study particular malignant processes (Rousseau-Merk *et al.*, 1982*a,b*), we grafted 60 embryonic tumours in order to correlate transplantability with histological group type, clinical data and relapse-free periods in the patient. Data on 13 other malignant paediatric tumours, and 12 benign tumours are also reported.

Tumour material was obtained by surgical excision. Histological types are described in Table I and comprise 60 embryonic tumours, 13 other childhood tumours and 12 benign ones. Forty-eight of the tumours were treated by radiotherapy and/or chemotherapy.

Nephroblastomas received either radiotherapy (20 Gy) or chemotherapy including the combination actinomycin D plus vincristine. Chemotherapy was given one month prior to excision of the primary tumours. In cases of relapse periods treatment varied according to the time of onset. Primary rhabdomyosarcomas were treated at various times prior to excision by chemotherapy (vincristine + actinomycin D + cyclophosphamide). Cases of relapse received the same chemotherapy with the addition of adriamycin and, in some cases, radiotherapy (45 Gy).

Male homozygous athymic nude mice of Swiss background were supplied by Iffa Credo (Lyon, France). Each tumour was subcutaneously inoculated (2 mm$^3$) usually in the flank of 3 nude mice aged 4–6 weeks. The mice were housed in plastic cages with air filter tops. Food, water, bedding, cages and tops were autoclaved prior to use.

Grafts were considered positive when tumour growth in the mice was clearly progressive and when the histology corresponded to that of the initial tumour.

Original and transplanted tumour samples were fixed in 15% formaldehyde dehydrated in 50 then 70 and 100% alcohol, immersed and embedded in paraffin. Sections (5 $\mu$m) were prepared and stained with H&E.

Because of the non-normal distribution of the values, non-parametric methods of analysis were preferred. Differences between groups for ordinal samples were tested using the chi-square method, and Fisher's exact probability test when the samples were small. Whitney's test was used for quantitative parameter comparisons between groups (Siegel, 1956).

The disease-free survival of patients was analyzed using life tables and compared using the log-rank test (Peto *et al.*, 1977).

Thirty-eight of the 73 malignant tumours (52%) were successfully xenografted as shown in Table I. The growth in nude mice was observed over a period from 3 weeks to 6 months.

The most successful grafts took in all 3 mice. False positive grafts were observed both with malignant and benign tumours where the tumour fragments grew very slowly for up to 6 months without exceeding 1 cm in diameter. These grafts, described as "stationary tumours" by Sharkey *et al.* (1978), were observed with 4 benign tumours (1 ganglioneuroma, 1 mature teratoma, 1 fibroma, 1 thymoma) and 6 malignant tumours (3 nephroblastomas, 1 corticosurrenaloma, 1 neuroblastoma and 1 malignant teratoma). They exhibited fibrous and/or differentiated histological features and could not be serially grafted from one mouse to another.

Correspondence: M.F. Rousseau-Merck.
Received 19 November 1984; and in revised form 22 April 1985.

© The Macmillan Press Ltd., 1985

Table I Histology, clinical data and growth of xenografted tumours

| Histological type | Positive take/No. of cases: Treated tumours | Untreated tumours | Percentage transplantability |
|---|---|---|---|
| (a) *Embryonic tumours* (60) | | | 48 |
| Nephroblastomas (34) | | | 47 |
| Primary | 6/19 | 5/9 | |
| Relapse | 5/6 | | |
| Rhabdomyosarcomas (13) | | | 100 |
| Primary | 1/1 | 4/4 | |
| Relapse | 8/8 | | |
| Neuroblastoma (10) | | | 0 |
| Primary | 0/7 | 0/3 | |
| Malignant teratoma | | | 0 |
| Primary | 0/2 | | |
| Relapse | 0/1 | | |
| (b) *Other childhood tumours* (13) | | | 69 |
| Hypercalcaemic primary renal tumours (3)[a] | | 3/3 | |
| Primary lymphoma (3) | | 2/3 | |
| Primary Ewing sarcoma (2) | 1/1 | 1/1 | |
| Miscellaneous[b] | 2/5 | | |
| Total malignant childhood tumours (73) | | | 52 |
| (c) *Benign tumours* (12)[c] | | 0/12 | 0 |

[a]Cases alreadly published (Rousseau-Merck *et al.*, 1982*a*, *b*).
[b]1/1 malignant histiocytosis [a], 1/1 mesothelioma (relapse), 0/1 corticosurrenaloma, 0/1 dysgerminoma, 0/1 Hodgkin's tumours.
[c]5 Eosinophilic granulomas, 2 ganglioneuromas, 1 thymoma, 1 neurofibroma, 1 desmoplastic fibroma, 1 hamartoma, 1 benign teratoma.

They are included as negative grafts in Table I. None of the other benign tumours gave growth in nude mice.

The take-rate was linked statistically to the histological type of the tumour ($P<0.001$) in the embryonic group.

All 13 rhabdomyosarcomas grew, the take-rate appearing to decline with increasing rhabdoid differentiation. While 100% transplantability was obtained with the 11 poorly differentiated rhabdomyosarcomas, each of the 2 more differentiated cases gave growth in only 1 of the 3 nude mice inoculated.

Neither poorly differentiated (neuroblastic) nor highly differentiated (ganglionic) neuroblastomas grew in nude mice. Five neuroblastomas were simultaneously transplanted into irradiated nude mice but none grew.

Forty-seven percent (16/34) of the nephroblastomas gave positive growth in nude mice. Predominantly blastomatous histological features appeared to be a favourable parameter for growth as opposed to fibroblastic and muscular elements. Only one of the 5 bilateral nephroblastomas produced a positive graft.

Every tumour grown in nude mice was histologically very similar to the tumour of origin and no maturation process could be distinguished in the first passage of the embryonic tumours. Only one case of lymphoma showed metastatic lymph node spread in the host. None of the other tumours gave distant metastases in the host.

Preoperative treatment of the tumours did not appear to interfere with transplantability ($P=0.57$) except for nephroblastomas. If we exclude the cases of relapse from the 34 nephroblastomas, the treated tumours produced more negative grafts than the untreated ones ($P<0.03$).

An important factor in the growth of the xenografts was the course of the malignant process (primary tumour or relapse). Fourteen of the 16 relapses were successfully grafted regardless of their histological type, which was significantly higher than the success rate obtained with the primary tumours ($P<0.001$). However, it should be noted that the number of relapses differed significantly depending in which histological group the embryonic tumours were classified ($P<0.0005$). The absence of relapse for the 10 neuroblastomas contrasted with the 8 relapses for the 13 rhabdomyosarcomas.

Fifty-three of the 73 patients with malignant tumours were followed clinically over a period ranging from 12 to 72 months after tumour excision and grafting. Clinical data obtained with 9 neuroblastomas showed a very poor course in only two children. By contrast, rapid relapse and death was observed in 11 of 12 patients with rhabdomyosarcoma, representing a particularly aggressive sample of that type of tumour. Positive growth of the graft was high when the relapse-free period following excision was low ($P<0.003$) (Figure 1).

**Figure 1** Disease free survival curves of 53 malignant cases. Curve (1) profiles the cases giving positive grafts and curve (2) the cases showing negative growth. (●) represent the cases with a poor clinical course (relapse) and (○) the cases remaining relapse free.

When metastatic samples were excluded and only the primary tumour samples were considered (41 cases) we obtained the same result ($P<0.007$). The latter was independent of the histological type since it was true of the nephroblastoma group ($P<0.05$) which was the only statistically sufficient sample of all the groups studied (Figure 2). The patient's age was not related to the transplantability of the tumour ($P=0.77$).

These results (52% transplantability of childhood tumours) were higher than the take rate of 35.7% observed by Sharkey *et al.* (1978) on 342 adult

**Figure 2** Disease free survival curves of 26 nephroblastoma cases. Symbols used are the same as for **Figure 1.**

tumours or the 36.5% observed by Neely *et al.* (1983) for 74 malignant paediatric tumours from a sample of 85. Variations in the transplantability of human tumours may be explained by variations in host characteristics, the methods of transplantation (Epstein *et al.*, 1976; Sharkey *et al.*, 1978; Hadju *et al.*, 1981), or by variations in the clinical or histological characteristics of the tumours themselves.

Since the sample size in this study was statistically sufficient the emphasis was placed on the characteristics of the main embryonic tumours with a view to defining the parameters determining transplantability rather than prognostic factors.

The transplantability of embryonic tumours was highly dependent on histological category: Within the same tumour type the take-rate increased as differentiation decreased. The high take-rate of rhabdomyosarcoma contrasted with the failure of growth of neuroblastoma. It is well known that most sarcomas can easily be grafted in nude mice (Sharkey *et al.*, 1978; Hadju *et al.*, 1981; Neely *et al.*, 1983). Attempts with rhabdomyosarcomas have been rare but Houghton *et al.* (1982) relate the successful transplantation of 7 out of 11 rhabdomyosarcomas grafted in immunodeprived mice.

The degree of histological differentiation does not explain the failure of grafting in neuroblastoma. Hata *et al.* (1984) have obtained 9 successes out of

36 neuroblastomas xenografted in nude mice. All 9 tumours were taken from patients over 22 months of age. In the other rare results with neuroblastoma the age of the patients was not given (Neely et al., 1983; Helson et al., 1975). The age parameter which is linked to the clinical course of the neuroblastoma (Einhorn, 1983) may explain our negative grafts since only 3 of the 10 patients with neuroblastomas were more than 2 years of age.

Histologically, all the grafts were similar to the tumour of origin. In a previous study we observed morphological and enzymatic modifications after serial passages of 8 malignant tumours in nude mice (Rousseau-Merck et al., 1984). All the modifications observed tended towards a loss of differentiation. One of these tumours showed complete histological transformation at the second passage and no longer appeared to be a nephroblastoma, but a lymphoma. The G6PD and GP1 isoenzyme patterns of this graft were of murine origin. It was the only case of a spontaneous murine tumour in the current series.

Preoperative chemotherapy and/or radiotherapy of primary tumours did not significantly affect the take-rate in nude mice except for nephroblastoma. Nephroblastomas are known to be cured with great efficency by chemotherapy (Lemerle et al., 1976). Secondary tumours occurring after therapy are easily grown in nude mice. In a series of childhood tumours, Neely et al. (1983) found no difference in transplantability between treated and untreated tumours although autopsy specimens grew remarkably well.

The disease-free survival curve showed a significant difference between the groups of patients with negative and those with positive tumour grafts.

The fact that some histological tumour types are closely linked to both the risk of relapse and positive grafting appears as a confounding factor. For this reason, the life table of the only statistically available histological tumour type (nephroblastoma) was studied but the difference between the 2 groups (negative and positive grafts) persisted ($P<0.05$) leading to the conclusion that good transplantability in nude mice is a poor prognostic factor in terms of the patient's clinical course.

In conclusion, all the poor prognostic factors related to the embryonic tumours such as histological tumour type, differentiation status, age of the patient for neuroblastoma, clinical state at the excision, are good prognostic factors for the transplantability of these tumours. Furthermore the high level of transplantability of sarcomas and rhabdomyosarcomas may provide a valuable screening tool for the selection of new therapeutic approaches to these tumours (Houghton et al., 1982).

We want to thank Dr. M. Guichard (Inserm U 247) for her help in handling irradiated nude mice, Prof. D. Pellerin and his staff for providing the tumours and Dr. M.F. Tournade for access to clinical data.

**References**

EINHORN, L. (1983). Are there factors preventing cancer development during embryonic life? *Oncodevelop. Biol. Med.*, **4**, 219.

EPSTEIN, A.L., HERMAN, N.M., KIM, H., DORFMAN, R.F. & KAPLAN, H.S. (1976). Biology of the human malignant lymphomas III. Intracranial heterotransplantation in the nude, athymic mouse. *Cancer*, **37**, 2158.

HADJU, S.I., LEMOS, L.B., KOZAKEWICH, H., HELSON, L. & BEATTIE, E. (1981). Growth pattern and differentiation of human soft tissue sarcomas in nude mice. *Cancer*, **47**, 90.

HATA, J., VEYAMA, Y., NOZI, H., TAMAOKI, N., AKATSUKA, A., SHIMIZU, K., MORIKAWA, Y. & SATO, K. (1984). Morphology and function of human neuroblastoma xenotransplanted in nude mice. *Cancer*, **53**, 2497.

HELSON, L., HELSON, C., RUBENSTEIN, R. & HADJU, S.I. (1977). Human neuroblastoma in nude mice. In *Proceedings of the Second International Workshop on Nude Mice* (eds. Nomura et al.). University of Tokyo Press, Tokyo. Gustav Fisher Verlag, Stuttgart.

HELSON, L. (1982). Pediatric tumor research with nude mice. In *The Nude Mouse in Experimental and Clinical Research vol. 2*, (Eds. Fogh & Giovanella) New York, Academic Press, Inc.

HOUGHTON, J.A., HOUGHTON, P.J. & WEBBER, B.L. (1982). Growth and characterization of childhood rhabdomyosarcomas as xenografts. *J. Natl Cancer Inst.*, **68**, 437.

LEMERLE, J., TOURNADE, M.F., GERARD-MARCHANT, R. & 7 others. (1976). Wilms' tumor: Natural history and prognostic factors. A retrospective study of 248 cases treated at the Institute Gustave Roussy 1952–1967, *Cancer*, **37**, 2557.

NEELY, J.E., BALLARD, E.T., BRITT, A.L. & WORKMAN, L. (1983). Characteristics of 85 pediatric tumors heterotransplanted into nude mice. *Exp. Cell. Biol.*, **51**, 217.

PETO, R., PIKE, M., ARMITAGE, P. & 7 others. (1977). Design and analysis of randomized clinical trials requiring prolonged observation of each patient. II Analysis and examples. *Br. J. Cancer*, **35**, 1.

ROUSSEAU-MERCK, M.F., JAUBERT, F., BACH, M.A., NIAUDET, P., COTTREAU, D. & NEZELOF, C. (1982*a*). Tumor cell line characterization of a malignant histiocytosis transplanted into nude mice. *Virchows Arch.* (Path. Anat.), **397**, 171.

ROUSSEAU-MERCK, M.F., BOCCON-GIBOD, L., NOGUES, C. & 5 others. (1982*b*). An original hypercalcemic infantile renal tumor without bone metastasis: Heterotransplantation to nude mice. *Cancer,* **50,** 85.

ROUSSEAU-MERCK, M.F., COTTREAU, D. & KAHN, A. (1984). Isozyme pattern in serially xenotransplanted childhood tumors. *Cancer Res.,* **44,** 1163.

SHARKEY, F.E,, FOGH, J., HADJU, S., FITZGERALD, P. & FOGH, J. (1978). Experience in surgical pathology with human tumor growth in the nude mouse in: *The Nude Mouse in Experimental and Clinical Research*, vol. 1. (Eds. Fogh & Giovanella) New York, Academic Press, Inc.

SIEGEL, SIDNEY (1956). *Non Parametric Statistics for the Behavorial Sciences.* McGraw-Hill Kogakusha, LTD, Tokyo.

WILLIS, R.A. (1962). *The Pathology of the Tumours of Children.* Oliver & Boyd, Edinburgh and London.